# 1320. In Vitro Activity of Lefamulin against *Staphylococcus aureus* Isolated from the Lower Respiratory Tract of Children with Cystic Fibrosis

**DOI:** 10.1093/ofid/ofab466.1512

**Published:** 2021-12-04

**Authors:** Helio S Sader, Susanne Paukner, Steven P Gelone, S J Ryan Arends, Rodrigo E Mendes

**Affiliations:** 1 JMI Laboratories, North Liberty, Iowa; 2 Nabriva Therapeutics GmbH, Vienna, Wien, Austria; 3 Nabriva Therapeutics US, Inc., King of Prussia, PA

## Abstract

**Background:**

Lefamulin is a first-in-class, oral and IV pleuromutilin antibiotic approved in the US, EU, and Canada for the treatment of community-acquired bacterial pneumonia (CABP) in adults. Lefamulin inhibits bacterial protein synthesis via a unique mechanism of action and its potency against *S. aureus* has been well established. We evaluated the *in vitro* activity of lefamulin against *S. aureus* from patients with cystic fibrosis (CF).

**Methods:**

Unique isolates (*n*=224) were collected from the lower respiratory tract (LRT) of children (≤17 years old) with CF and LRT infection. Organisms were from qualified respiratory specimens and determined to be the probable cause of infection by the participant center. The isolates were collected in 2018-2020 from 22 medical centers in 11 countries and tested by broth microdilution methods at JMI Laboratories. Most isolates were from the US (43.3%), Spain (24.1%), France (20.5%), and Costa Rica (7.1%).

**Results:**

Lefamulin was highly active against the CF *S. aureus* collection (MIC_5090_, 0.06/0.12 mg/L), with 99.6% of isolates inhibited at ≤0.25 mg/L, consistent with the susceptible [S] breakpoint published by the US FDA, CLSI, and EUCAST. Only 1 lefamulin-non-S (MIC, 1 mg/L) isolate was observed, a methicillin-susceptible (MSSA) collected in Costa Rica in 2018 and carrying a *vga*(A) gene. Lefamulin retained potent activity against methicillin-resistant (R) *S. aureus* (MRSA, n=52; MIC_50/90_, 0.06/0.12 mg/L), azithromycin-R (n=115; MIC_50/90_, 0.06/0.12 mg/L), levofloxacin-R (n=23; MIC_50/90_, 0.06/0.12 mg/L), clindamycin-R (n=11; MIC_50/90_, 0.06/0.12 mg/L), and gentamicin-R (n=9; MIC range of 0.03-0.12 mg/L) isolates as well as those isolates with multiple resistance phenotypes. Against MRSA, susceptibility to azithromycin was 23.5% and to levofloxacin 64.7%. All isolates were susceptible to vancomycin, linezolid and ceftaroline (Table). Among isolates from the US (n=97), the MRSA rate was 30.9% and all isolates were Lefamulin-S (MIC_5090_, 0.06/0.12 mg/L).

**Conclusion:**

Lefamulin demonstrated potent *in vitro* antibacterial activity against *S. aureus* from children with CF, regardless of resistance phenotype. Lefamulin may represent a valuable treatment option for CF patients with *S. aureus* LRT infections.

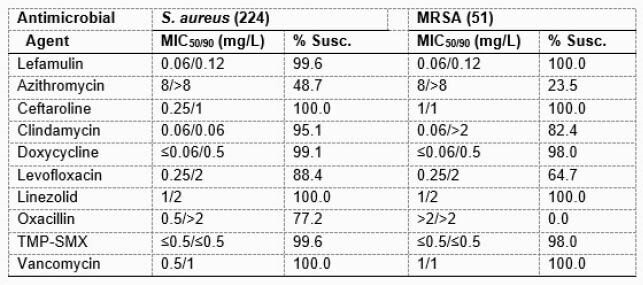

**Disclosures:**

**Helio S. Sader, MD, PhD, FIDSA**, **AbbVie (formerly Allergan**) (Research Grant or Support)**Basilea Pharmaceutica International, Ltd.** (Research Grant or Support)**Cipla Therapeutics** (Research Grant or Support)**Cipla USA Inc.** (Research Grant or Support)**Department of Health and Human Services** (Research Grant or Support, Contract no. HHSO100201600002C)**Melinta Therapeutics, LLC** (Research Grant or Support)**Nabriva Therapeutics** (Research Grant or Support)**Pfizer, Inc.** (Research Grant or Support)**Shionogi** (Research Grant or Support)**Spero Therapeutics** (Research Grant or Support) **Susanne Paukner, PhD**, **Nabriva Therapeutics GmbH** (Employee) **Steven P. Gelone, PharmD**, **Nabriva Therapeutics US, Inc.** (Employee) **S J Ryan Arends, PhD**, **AbbVie (formerly Allergan**) (Research Grant or Support)**GlaxoSmithKline, LLC** (Research Grant or Support)**Melinta Therapeutics, LLC** (Research Grant or Support)**Nabriva Therapeutics** (Research Grant or Support)**Spero Therapeutics** (Research Grant or Support) **Rodrigo E. Mendes, PhD**, **AbbVie** (Research Grant or Support)**AbbVie (formerly Allergan**) (Research Grant or Support)**Cipla Therapeutics** (Research Grant or Support)**Cipla USA Inc.** (Research Grant or Support)**ContraFect Corporation** (Research Grant or Support)**GlaxoSmithKline, LLC** (Research Grant or Support)**Melinta Therapeutics, Inc.** (Research Grant or Support)**Melinta Therapeutics, LLC** (Research Grant or Support)**Nabriva Therapeutics** (Research Grant or Support)**Pfizer, Inc.** (Research Grant or Support)**Shionogi** (Research Grant or Support)**Spero Therapeutics** (Research Grant or Support)

